# Characterization of Antileishmanial Compounds from *Lawsonia inermis* L. Leaves Using Semi-High Resolution Antileishmanial Profiling Combined with HPLC-HRMS-SPE-NMR

**DOI:** 10.3389/fphar.2017.00337

**Published:** 2017-05-31

**Authors:** Kashif Iqbal, Javeid Iqbal, Dan Staerk, Kenneth T. Kongstad

**Affiliations:** ^1^Department of Pharmacology, Faculty of Pharmacy and Health Sciences, University of BalochistanQuetta, Pakistan; ^2^Department of Drug Design and Pharmacology, Faculty of Health and Medical Sciences, University of CopenhagenCopenhagen, Denmark

**Keywords:** *Leishmania tropica*, *Lawsonia inermis*, leishmaniasis, semi-high-resolution inhibition profile, HPLC-HRMS-SPE-NMR

## Abstract

This work describes an analytical platform based on semi-high-resolution antileishmanial profiling combined with hyphenation of high-performance liquid chromatography – high-resolution mass spectrometry – solid-phase extraction – nuclear magnetic resonance spectroscopy, i.e., *semi*HR-antileishmanial assay/HPLC-HRMS-SPE-NMR. The platform enables fast pinpointing of HPLC peaks representing *Leishmania tropica* inhibitors in complex matrices, with subsequent structural identification of targeted inhibitors. Active analytes were cumulatively trapped on SPE cartridges and the structures elucidated by analysis of NMR spectra obtained in the HPLC-HRMS-SPE-NMR mode. This led to the identification of six known compounds 2,4,6-trihydroxyacetophenone-2-*O*-β-D-glucopyranoside (**1**), lalioside (**2**), luteolin-4′-*O*-β-D-glucopyranoside (**3**), apigenin-4′-*O*-β-D-glucopyranoside (**4**), luteolin (**5**), and apigenin (**6**). IC_50_ of the active compounds were determined with luteolin being the most potent inhibitor with an IC_50_ value of 4.15 μg/ml. The platform proved to be an efficient method for the identification of *L. tropica* inhibitors.

## Introduction

Leishmaniasis, caused by parasites belonging to the genus *Leishmania* (Family Trypanosomatidae), is a major public health problem in tropical and sub-tropical regions. The parasite is transmitted by the sand fly vector, with dogs, sheep, rats, horses, and cats being common animal hosts of leishmaniasis (Pearson and Sousa, 1996; Markle and Makboul, 2004). WHO has reported that people from 98 countries – covering five continents – are at high risk of contracting leishmaniasis, and it is estimated that approximately 12 million people are currently infected (Alvar et al., 2012). Cutaneous leishmaniasis is caused by different *Leishmania* species, e.g., *Leishmania tropica, Leishmania major, Leishmania amazonensis*, and *Leishmania braziliensis*. In Pakistan, *L. tropica* is the main cause of cutaneous leishmaniasis (Rowland et al., 1999; Brooker et al., 2004). First line therapy for cutaneous leishmaniasis in Europe, Asia, and Africa is pentavalent antimonials, i.e., sodium stibogluconate and meglumine antimoniate (Markle and Makboul, 2004). However, antimonials have severe side effects like myalgia, pancreatitis, cardiac arrhythmia, hepatitis, and accumulation of the drug in liver and spleen. Thus, there is an urgent need for new chemical entities for non-toxic and effective treatment of leishmaniasis (Brooker et al., 2004; Markle and Makboul, 2004).

*Lawsonia inermis* L. is commonly known as Henna or Mehndi (Family Lythraceae) (Kumar et al., 2005). It is native to Northern Africa and South-western Asia, and is cultivated in many tropical and sub-tropical regions (Cartwright-Jones, 2006). In Pakistan, it is widely found in the Dera Ismail khan and Bannu districts of the Khyber Pakhtunkhwa province. *L. inermis* is extensively used for different medicinal purposes, and possess a variety of biological and pharmacological activities, including antioxidant (Dasgupta et al., 2003), antibacterial (Ali et al., 2001), antifungal (Singh and Pandey, 1989), antiviral (Khan et al., 1991), antiparasitic (Okpeton et al., 2004), analgesic (Mohsin et al., 1989), cytotoxic (Ali and Grever, 1998), antidiabetic (Arayne et al., 2007), antileishmanial (Iqbal et al., 2016a,b) and protein glycation inhibitory activity (Sultana et al., 2009). *L. inermis* is chemically well investigated (Semwal et al., 2014), and more than 135 compounds have been reported from the genus. This includes phenolic compounds [flavonoids (Liou et al., 2013), naphthalenes (Hsouna et al., 2011), naphthoquinones (Almeida et al., 2012), coumarins (Chaudhary et al., 2010), alkylphenones (Hsouna et al., 2011)], terpenes [volatile terpenes (Hema et al., 2010), non-volatile terpenes (Liou et al., 2013)], aliphatic hydrocarbons, and alkaloids (Iqbal et al., 2016b).

A major bottleneck in our ongoing search for antiparasitic constituents from plants (Sairafianpour et al., 2002; Ziegler et al., 2002; Pedersen et al., 2009) has been the traditional time-consuming bioassay-guided isolation of the antileishmanial compounds. This urged us to implement new bioanalytical technologies for faster analyses targeting the bioactive constituents. Hyphenation of separation techniques, spectroscopic methods, and bioassays has in recent years proven to be an efficient strategy for this purpose (Van Beek et al., 2009). While the commonly used hyphenation of high-performance liquid chromatography coupled with high-resolution mass spectrometry (HPLC-HRMS) is a fast and sensitive technique, it has several limitations when it comes to full structural elucidation of complex natural products. Especially promising is therefore the additional hyphenation of HPLC and HPLC-HRMS with solid-phase extraction and nuclear magnetic resonance spectroscopy, i.e., HPLC-(HRMS)-SPE-NMR (Lambert et al., 2005; Johansen et al., 2011), which allows full structural identification of complex natural products directly from crude extracts. As HPLC-HRMS-SPE-NMR only allows for chemical analysis, the recent combination with semi-high-resolution and/or high-resolution bioactivity profiling, i.e., microfractionation in microplates followed by bioassaying to yield a semi-high or high-resolution inhibition profile, is one of the most promising technological developments within bioanalytical plant research. The resulting HR-bioassay/HPLC-HRMS-SPE-NMR technology platform has already proven effective for fast identification of α-glucosidase inhibitors (Schmidt et al., 2012, 2014; Kongstad et al., 2015; Wubshet et al., 2015), aldose reductase inhibitors (Tahtah et al., 2015), α-amylase inhibitors (Okutan et al., 2014), and radical scavengers (Wiese et al., 2013; Wubshet et al., 2013; Liu et al., 2015) directly from crude plant extracts. However, the current work is the first example of semi-high-resolution antileishmanial inhibition profiling coupled with HPLC-HRMS-SPE-NMR.

## Materials and Methods

### Chemicals

Fetal bovine serum (FBS), dimethyl sulfoxide (DMSO), RPMI-1640 medium, Amphotericin B, penicillin, streptomycin, formic acid, analytical grade HPLC solvents (chloroform, methanol, ethyl acetate, *n*-hexane, acetone), and methanol-*d*_4_ (99.8 atom % of deuterium) were purchased from Sigma-Aldrich (St. Louis, MO, United States), whereas silica gel 60 0.063–0.200 mm (70–230 mesh ASTM) was purchased from MERCK (Darmstadt, Germany). Water used for HPLC was purified by deionization and 0.22 μm membrane filtration (Millipore, Billerica, MA, United States).

### Collection of Plant Material and Preparation of Crude Extract

Leaves of *Lawsonia inermis* L. were collected from the territory of Dera Ismail Khan, Khyber Pakhtunkhwa (KPK), Pakistan in August and September 2014. Identification was performed by Dr. Siraj-ud-Din, Department of Botany, and a voucher specimen [accession number: Bot, 200101 (pup)] was deposited at Department of Botany, University of Peshawar (UOP), KPK. The leaves were washed with distilled water before drying in the shade at temperatures below 35°C. The leaves were stored in a cool dark place until use. Ground material of *L. inermis* (leaves, 1 kg) was extracted with methanol (2 L) for 1 week with regular stirring. The extract was filtered and concentrated in vacuo to afford 500 g of an oily extract.

### Preparative-Scale Fractionation

The crude leaves extract (250 g) was fractionated by means of silica gel column chromatography (100 cm × 50 mm i.d column, silica gel 60 0.063–0.200 mm from MERCK (Darmstadt), Germany). A gradient elution was used starting with *n*-hexane-chloroform (99:1) with 20% step-wise increments to 100% chloroform, followed by 20% increments of ethyl acetate, and subsequent 20% increments of methanol to 100% methanol. Each of the increments constituted 500 mL solvent, which yielded 89 fractions. Based on TLC, fractions 1–5 were pooled (fraction F1, 6 g), fractions 6–25 (F2, 9.5 g), fractions 26–40 (F3, 3.5 g), fractions 41–45 (F4, 5 g), fractions 46–65 (F5, 10 g), fractions 66–80 (F6, 6 g), and fractions 81–89 (F7, 8.5 g). Fraction F1–F7 was subjected for *in vitro* antileishmanial activity, *vide infra*.

### *In Vitro* Antileishmanial Activity

*In vitro* antileishmanial activity of *L. inermis* fractions were performed with clinically isolated *L. tropica* promastigotes (KWH23, Recently Pakistani clinically isolated strain, UOP, Pakistan). The *in vitro* antileishmanial growth inhibition assay was adopted from Iqbal et al. (2016b). Promastigotes of *L. tropica* were cultured in RPMI-1640 medium containing 10% FBS, 200 U/mL of penicillin, and 0.2 mg/mL of streptomycin. The parasites were cultured at 26°C for 4 days in an incubator (Gallenkamp, Size 1, United Kingdom), where after promastigotes were harvested in sterile tubes. The number of promastigotes was measured by transferring 5–10 μL to a haemocytometer (Reichert Technologies, Depew, NY, United States), and counting the number of promastigotes under upright microscope (CX31, Olympus, Tokyo, Japan). The viable cell count was calculated using the formula:

$$Viable cell count\;\left(\mathrm{live}\;\mathrm{cells}/\mathrm{mL}\right)=\frac{Number of live cells counted}{Number of large corner squares counted}\times \mathrm{Dilution}\times 10,000$$

The harvested promastigotes were subsequently centrifuge at 4°C at 2000 rpm for 10 min, the supernatant removed, and the pellet reconstituted in fresh RPMI-1640 medium with 10% FBS to obtain a concentration of 1.4 × 10^6^ promastigotes/mL which was distributed in a 96 well culture plate (180 μL each) and incubated for 2 days at 26°C with fractions of *L. inermis*.

The percentage inhibition of parasite growth was calculated as the mean of three replicate measurements with standard deviation using the equation:

$$Percentage inhibition=\frac{count of control promastigotes-count of treated promastigotes}{count of control promastigotes}\times 100$$

IC_50_ values were determined by non-linear regression analysis using Graph Pad Prism 6 software.

### Preparative-Scale Fractionation of F5

An injection solution of 0.1 g/mL of fraction F5 was subjected to preparative-scale RP-HPLC using an Agilent 1100 system equipped with two preparative solvent delivery units, a multiple wavelength detector, an autosampler, and a fraction collector. Separation was performed using a 250 mm × 21.2 mm i.d. Phenomenex Luna C_18_ column with 5 μm particle size (Phenomenex, Torrance, CA, United States) operated at room temperature. The aqueous eluent (A) consisted of water–acetonitrile (95:5), and the organic eluent (B) consisted of water–acetonitrile (5:95) both acidified with 0.1% formic acid. The eluent flow rate was maintained at 20 mL/min. Repeated injection (3 × 900 μL) of the above solution was followed by a gradient elution profile as follows: 0 min, 10% B; 40 min, 40% B; 45 min, 100% B; 55 min, 100% B; 56 min, 10% B. This afforded 27.1 mg of subfraction 1, 10.9 mg of subfraction 2, 9.3 mg of subfraction 3, 8.3 mg of subfraction 4, 15.2 mg of subfraction 5, 10.0 mg of subfraction 6, 10.3 mg of subfraction 7, 6.8 mg of subfraction 8, 21.1 mg of subfraction 9, 5.3 mg of subfraction 10, 6.9 mg of subfraction 11, 5.3 mg of subfraction 12, 3.4 mg of subfraction 13, 2.5 mg of subfraction 14, 4.4 mg of subfraction 15, 96.6 mg of subfraction 16, 35.1 mg of subfraction 17, 3.9 mg of subfraction 18, 6.3 mg of subfraction 19, and 3.7 mg of subfraction 20. All collected subfractions were concentrated under reduced pressure at 45°C in rotary evaporator. Initially, the subfractions were reconstituted in 0.1% DMSO in order to assess the IC_50_ values in the *in vitro* antileishmanial growth inhibition assay, *vide supra*.

### HPLC-HRMS-SPE-NMR

The HPLC-HRMS-SPE-NMR system consisted of an Agilent 1200 chromatograph comprising of a quaternary pump, degasser, thermostated column compartment, auto sampler, and photodiode array detector (Santa Clara, CA, United States), a Bruker microOTOF-Q II mass spectrometer (Bruker Daltonik, Bremen, Germany) equipped with an electrospray ionization source and operated via a 1:99 flow splitter, a Knauer Smartline K120 pump for post-column dilution (Knauer, Berlin, Germany), a Spark Holland Prospekt2 SPE unit (Spark Holland, Emmen, Netherlands), a Gilson 215 liquid handler equipped with a 1 mm needle for automated filling of 1.7 mm NMR tubes, and a Bruker Avance III 600 MHz NMR spectrometer (^1^H operating frequency 600.13 MHz) equipped with a Bruker Sample Jet sample changer and a cryogenically cooled gradient inverse triple-resonance 1.7 mm TCI probe-head (Bruker Biospin, Rheinstetten, Germany). Mass spectra were acquired in positive and negative ion modes, using drying temperature of 200°C, capillary voltage of -4100 and +4000 V for positive and negative ion modes, respectively, nebulizer pressure of 2.0 bar, and drying gas flow of 7 L/min. The negative ion mode HPLC-HRMS analysis was performed in a different experiment using identical chromatographic condition. A solution of sodium formate clusters was injected in the beginning of each run to enable internal mass calibration. Chromatographic separation was acquired on a Phenomenex Luna C_18_(2) column (150 mm × 4.6 mm, 3 μm, 100 Å; Phenomenex, Torrance, CA, United States) maintained at 40°C, using water–acetonitrile (95:5) (eluent A) and acetonitrile–water (95:5) (eluent B), both acidified with 0.1% formic acid. At a flow rate of 0.5 mL/min the following gradient elution profile was used: 0 min, 10% B; 40 min, 40% B; 45 min, 100% B; 55 min, 100% B; 56 min, 10% B. Cumulative SPE trapping of peaks 1–9 of *L. inermis* fraction F5 was performed after 10 consecutive separations. The HPLC eluate was diluted with Milli-Q water at a flow rate of 1.5 mL/min prior to trapping on 10 mm × 2 mm i.d. resin GP (general purpose, 5–15 μm, spherical shape, polydivinyl-benzene phase) SPE cartridges from Spark Holland (Emmen, Netherlands), and analytes were trapped using absorption thresholds (280 and 330 nm). SPE cartridges were conditioned with 1000 μL of acetonitrile at 6 mL/min and equilibrated with 500 μL of Milli-Q water at 1 mL/min prior to trapping. Loaded cartridges were dried with pressurized nitrogen gas for 45 min each prior to elution with methanol-*d*_4_. Separations were controlled by Bruker HyStar version 3.2 software, automated filling of NMR tubes was controlled by Prep Gilson ST version 1.2 software, and automated NMR acquisition was controlled by Bruker IconNMR version 4.2 software. NMR data processing was performed using Bruker Topspin version 3.2 software.

### NMR Experiments

All NMR spectra were recorded in methanol-*d*_4_ at 300 K. ^1^H and ^13^C chemical shifts were referenced to the residual solvent signal (δ 3.31 and 49.00, respectively). One dimensional ^1^H NMR spectra were with 30° pulses, 3.66 s interpulse intervals, 64k data points, and multiplied with an exponential function corresponding to line-broadening of 0.3 Hz prior to Fourier transform. Phase-sensitive DQF-COSY spectra were recorded using a gradient-based pulse sequence with a 20 ppm spectral width and 2k × 256 data points (processed with forward linear prediction to 1k data points). Multiplicity-edited HSQC spectra were acquired with the following parameters: spectral width 20 ppm for ^1^H and 165 ppm for ^13^C, 2k × 256 data points (processed with forward linear prediction to 1k data points), and 2.0 s relaxation delay. HMBC spectra were optimized for ^n^*J*_C,H_ = 10 Hz and acquired using the following parameters: spectral width 20 ppm for ^1^H and 222 ppm for ^13^C, 2k × 128 data points (processed with forward linear prediction to 1k data points), and 1.5 s relaxation delay.

## Results

The current work describes the use of HPLC-HRMS-SPE-NMR for dereplication of metabolites in extract of *Lawsonia inermis* leaves – in combination with semi-high-resolution antileishmanial profiling. Initially, crude methanol extract was tested for its ability to inhibit extracellular promastigotes of *L. tropica* at concentrations of 100, 50, 25, and 10 mg/ml, which resulted in 100% inhibition at all concentrations. The crude methanol extract was subsequently subjected to preparative-scale column chromatography for separation into seven fractions (F1–F7). These fractions were tested for their ability to inhibit extracellular promastigotes, and as seen in **Table 1**, Fraction 5 showed the strongest antileishmanial activity.

**Table 1:** IC_50_ values of *Lawsonia inermis* leaves extract and fractions.

Sample	Sample concentrations (mg/ml)	Percent inhibition
F1	10	6.25 ± 0.57
	25	18.75 ± 1.00
	50	31.25 ± 0.57
	100	37.5 ± 0.57
F2	10	7.50 ± 0.00
	25	22.5 ± 0.57
	50	30.0 ± 1.00
	100	35.0 ± 0.00
F3	10	1.25 ± 0.57
	25	6.25 ± 0.00
	50	25.0 ± 0.57
	100	27.5 ± 0.57
F4	10	6.25 ± 0.57
	25	7.50 ± 0.57
	50	12.5 ± 0.57
	100	18.75 ± 0.57
F5	10	98.0 ± 0.57
	25	98.0 ± 0.57
	50	99.0 ± 0.00
	100	100 ± 0.00
F6	10	12.5 ± 0.57
	25	25.0 ± 1.15
	50	37.5 ± 0.57
	100	43.75 ± 0.57
F7	10	0.00 ± 0.00
	25	12.5 ± 0.57
	50	18.75 ± 0.00
	100	25.0 ± 0.57

*Data represent mean percent inhibition ± SD of three replicates.*

Fraction 5 was selected for semi-high-resolution antileishmanial screening with the eluate from 10 to 40 min being separated into 20 sub-fractions in 96-well microplates. The eluate was evaporated, and the dried material in each well was redissolved in DMSO to make stock and working solutions. These solutions were assayed for their *in vitro* antileishmanial activity toward extracellular promastigotes. The active major constituents of subfractions 5, 7, 10, and 13 were identified by HPLC-HRMS-SPE-NMR directly from the crude extract and the IC_50_ values of the identified, active metabolites were determined.

## Discussion

### HPLC-HRMS-SPE-NMR

**FIGURE 1:**
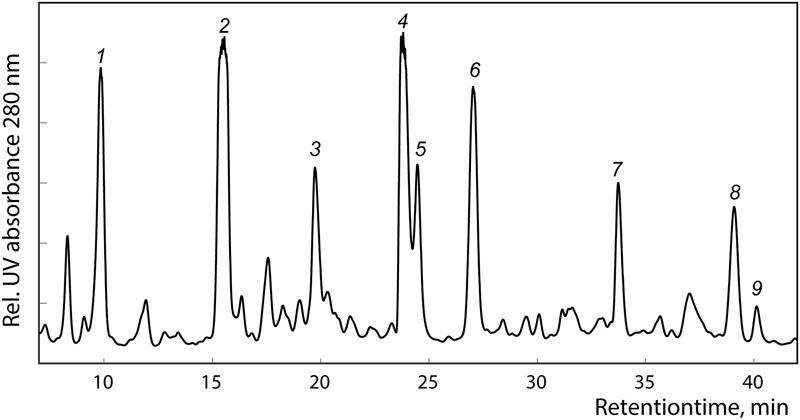
HPLC chromatogram of *Lawsonia inermis* L. leaves F5 extract at 280 nm acquired in the HPLC-HRMS-SPE-NMR mode.

**FIGURE 2:**
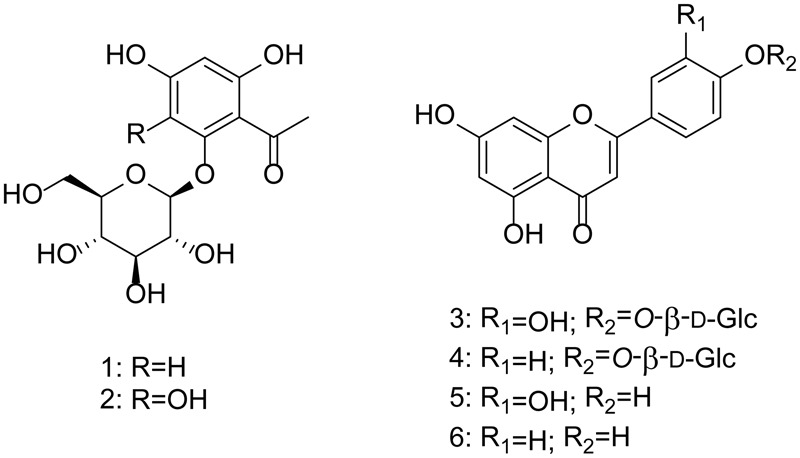
Compounds identified from *Lawsonia inermis* L. leaves extract.

The major metabolites of the active fractions (peaks *2, 4, 5, 7*, and *9*), together with additional four peaks, were subjected to HPLC-HRMS-SPE-NMR analysis (**Figure 1**). Detailed HRMS and NMR analysis resulted in identification of six known compounds **1–6** (**Figure 2**) corresponding to peaks *1, 2, 4, 5, 7*, and *9*, while for peaks *3, 6*, and *8*, the quality of the NMR data did not allow for full structure elucidation. ^1^H NMR and HRMS data of **1–6** obtained in the HPLC-HRMS-SPE-NMR mode is provided in the compound summary paragraph.

The known compounds were identified by comparison of their spectral data with those reported in the literature as 2,4,6-trihydroxyacetophenone-2-*O*-β-D-glucopyranoside (**1**) (Lee et al., 1996), lalioside (**2**) (Takeda and Fatope, 1988), luteolin-4′-*O*-β-D-glucopyranoside (**3**) (Lee et al., 2002), apigenin 4′-*O*-β-D-glucopyranoside (**4**) (Ding et al., 2004), luteolin (**5**) (Scognamiglio et al., 2014), and apigenin (**6**) (Miyazawa and Hisama, 2003) and confirmed by 2D NMR analysis.

### Antileishmanial Activity

In our work, F5 was subjected to prep HPLC for time slice based fractionation. It results in 20 sub-fractions which were further analyzed by extracellular promastigotes in which four sub-fractions showed promising inhibitory activity as shown in **Table 2**. After observation of their significant antileishmanial activity in the sub-fraction screening, lalioside (**2**), luteolin-4′-*O*-β-D-glucopyranoside (**3**), apigenin 4′-*O*-β-D-glucopyranoside (**4**), luteolin (**5**), and apigenin (**6**) were isolated by preparative- and analytical-scale HPLC, and the materials were used for assessing IC_50_ values in antileishmanial activity assay.

**Table 2:** IC_50_ values of major metabolites in active *Lawsonia inermis* L. subfractions.

Compound (*subfraction*)	IC_50_ value (μg/mL)
Lalioside (*SF 5*)	5.02
Luteolin-4′-*O*-β-D-glucopyranoside (*SF 7*)	10.27
Apigenin-4′-*O*-β-D-glucopyranoside (*SF 7*)	9.51
Luteolin (*SF 10*)	4.15
Apigenin (*SF 13*)	8.30
Amphotericin B (*Positive control*)	1.17

*Data represent mean IC_*50*_ values with 95% confidence intervals.*

This is in agreement with previously observed inhibition of *L. tropica* by crude extract of different *L. inermis* plant parts (Iqbal et al., 2016b). Tasdemir et al. (2006) have previously investigated the structure-activity relationship of flavonoids, including luteolin (**5**) and apigenin (**6**). It was found that a minimum of two hydroxy substituents on the A ring, preferably on position 5, 7, and 8, greatly increased the antileishmanial potential. Furthermore, the double bond between C-2 and C-3 proved to be important for the activity. Both luteolin (**5**) and apigenin (**6**) are di-substituted on the ‘A’ ring on positions 5 and 7 and both having the important double bond Δ^2,3^ accounting for the IC_50_ values of 4.15 and 8.30 μg/mL, respectively, with the differences in the antileishmanial potential attributes to the different substitution pattern on the B ring. Interestingly, the 4′-*O*-β-D-glucosides of **5** and **6** (10.27 and 9.51 μg/mL, **3** and **4,** respectively) showed a markedly decreased inhibitory effect against extracellular promastigotes. This is in line with previous reports that glucosylation on the A ring have similar effects (Tasdemir et al., 2006). Lalioside (**2**), a tetra-substituted acetophenone glucoside, showed promising inhibitory effects with an IC_50_ value of 5.02 μg/mL while the tri-substituted acetophenone glucoside (**1**) did not show noteworthy antileishmanial potential in the semi-high-resolution screening, indicating the importance of the C-3 hydroxyl substituent. While both luteolin (**5**) and apigenin (**6**) have previously been shown to possess activity against *L. donovani* (0.8 and 1.8 μg/mL, respectively) (Tasdemir et al., 2006) this is the first report of their *in vitro* activity against *L. tropica* and the first time lalioside (**2**), luteolin-4′-*O*-β-D-glucopyranoside (**3**), and apigenin-4′-*O*-β-D-glucopyranoside (**4**) are reported against any *Leishmania* species.

## Conclusion

In conclusion, luteolin and lalioside are potent inhibitors while apigenin, apigenin-4′-*O*-β-D-glucopyranoside, and luteolin-4′-*O*-β-D-glucopyranoside have moderate inhibitory effect on extracellular promastigotes of *L. tropica*. Further studies on identified compounds of *Lawsonia Inermis* are planned to investigate specificity and structure-activity relationship.

## Author Contributions

KI: Performed assay and chemical investigation. Participated in writing the manuscript. JI: Supervised and assisted KI on the antileishmanial work and participated in writing the manuscript. DS: Supervised and assisted KI on the chemical investigation and participated in writing the manuscript. KK: Took part in the chemical investigation and participated in writing the manuscript.

## Conflict of Interest Statement

The authors declare that the research was conducted in the absence of any commercial or financial relationships that could be construed as a potential conflict of interest.
